# Partnering with patients in quality improvement: towards renewed practices for healthcare organization managers?

**DOI:** 10.1186/s12913-019-4618-8

**Published:** 2019-11-08

**Authors:** Nathalie Clavel, Marie-Pascale Pomey, Djahanchah Philip (Sacha) Ghadiri

**Affiliations:** 1 Department of Health Policy, Management and Evaluation, School of Public Health, University of Montreal, Montreal, Canada; 2 Department of Management, HEC Montreal, Montreal, Canada

**Keywords:** Patient partnership, Quality improvement, Managerial practices, Healthcare organization managers, Implementation of change

## Abstract

**Background:**

Around the world, many healthcare organizations engage patients as a quality improvement strategy. In Canada, the University of Montreal has developed a model which consists in partnering with patient advisors, providers, and managers in quality improvement. This model was introduced through its Partners in Care Programs tested with several quality improvement teams in Quebec, Canada. Partnering with patients in quality improvement brings about new challenges for healthcare managers. This model is recent, and little is known about how managers contribute to implementing and sustaining it using key practices.

**Methods:**

In-depth multi-level case studies were conducted within two healthcare organizations which have implemented a Partners in Care Program in quality improvement. The longitudinal design of this research enabled us to monitor the implementation of patient partnership initiatives from 2015 to 2017. In total, 38 interviews were carried out with managers at different levels (top-level, mid-level, and front-line) involved in the implementation of Partners in Care Programs. Additionally, seven focus groups were conducted with patients and providers.

**Results:**

Our findings show that managers are engaged in four main types of practices: 1-designing the patient partnership approach so that it makes sense to the entire organization; 2-structuring patient partnership to support its deployment and sustainability; 3-managing patient advisor integration in quality improvement to avoid tokenistic involvement; 4-evaluating patient advisor integration to support continuous improvement. Designing and structuring patient partnership are based on typical management practices used to implement change initiatives in healthcare organizations, whereas managing and evaluating patient advisor integration require new daily practices from managers. Our results reveal that managers at all levels, from top to front-line, are concerned with the implementation of patient partnership in quality improvement.

**Conclusion:**

This research adds empirical support to the evidence regarding daily managerial practices used for implementing patient partnership initiatives in quality improvement and contributes to guiding healthcare organizations and managers when integrating such approaches.

## Background

Internationally, patient engagement has become central to healthcare quality improvement efforts [1–5]. Several healthcare institutions, including the World Health Organization [6], the Institute of Medicine [7], and the Institute for Healthcare Improvement [8], promote patient engagement as a promising strategy to enhance healthcare quality and safety. Within healthcare organizations (HCOs), patients and their families can be engaged in quality improvement (QI) activities and structures [9, 10]. Engaging patients in QI is a way to bridge the gap between the quality that patients expect, and the intended quality as traditionally defined by managers and providers. Many HCOs have started to engage patients as part of their QI activities and structures [11, 12]. In Canada, this movement has expanded ever since accreditation bodies and governments defined new guidelines, standards and policies that make patient engagement a core strategy in achieving higher quality in healthcare settings [13–15].

In HCOs, several models exist to integrate the perspective of patients into QI. First, patient engagement occurs in varying degrees: consultation, collaboration and co-construction or partnership [9, 10]. Patients can be engaged in different activities related to QI: defining quality criteria for care and services [16]; co-designing care processes [17] and developing projects to improve the quality of care and services [18]; providing feedback on the quality of care and services [19]. Second, patient engagement in QI can take place within interdisciplinary QI clinical teams and managerial committees or projects [12].

In Canada, several interdisciplinary clinical teams within different HCOs have implemented initiatives based on partnering with patients for QI purposes and little is known about the practices used to implement them in different organizational and clinical settings [12, 20–22]. Studies on patient partnership (PP) broadly focus on identifying contextual and organizational factors associated with the implementation of PP, without understanding what managers actually do in practice at different levels of HCOs [19]. Partnering with patients in QI introduces new challenges within HCOs. The integration of a new actor, “the patient advisor” (PA) within QI teams, is challenging and can result in tokenistic involvement without real contribution from the PA [12]. In addition, as with any innovative initiative introduced within HCOs, PP in QI requires special efforts on the part of managers to support its deployment and sustainability.

Hence, the main goal of this research is to study key managerial practices to implement PP in QI and has two main objectives: 1-describe the implementation of a PP program in two different clinical areas; 2-identify managerial practices at different management levels used to implement PP in QI.

## Methods

### Approach

We conducted multi-level case studies within two HCOs in Canada (Quebec) that have implemented the Partners in Care Program. A case study research method is particularly appropriate for studying poorly understood, complex and process-related interventions, such as the implementation of PP initiatives because it is based on an in-depth understanding of the context of the intervention [23, 24]. The longitudinal design of this research enabled us to monitor the implementation of PP from 2015 to 2017. Qualitative methods were used to collect and analyze data (see Data collection and Data analysis sections). We used the Standards for Reporting Qualitative Research (SRQR) to report our research results [25].

#### Case selection

The cases correspond to recently (< 2 years, at the time of the first data collection period) implemented Partners in Care Programs in clinical teams within Canadian HCOs that aim at partnering with patients in QI. The Collaboration and Patient Partnership Unit at the University of Montreal’s (UofM) Faculty of Medicine [26] developed the Partners in Care Program, tested with several interdisciplinary QI teams within different HCOs [10, 18]. This program aims to introduce PAs into QI committees. PAs are volunteers who share their experiential knowledge with providers and managers to provide direct input on care and services [12]. A QI committee works according to a Plan-Do-Study-Act method based on improvement cycles. The committee is supervised by a program manager and the team’s medical chief and has one or two institutional collaboration leaders (ICLs). ICLs are providers or managers, external to the team, who are responsible for supporting PA integration. Finally, a patient coach, with prior PA experience, is assigned to newly integrated PAs.

The two cases were selected based on the most-different case selection procedure described by Gerring [27]. Specifically, we followed a maximum variance sample strategy [28] to choose cases with the following characteristics: occurring in different HCOs and locations (urban vs. rural) and within different clinical settings (mental health and oncology). Aside from the above differences, Partners in Care Programs were implemented identically in both cases, with methodological support from the UofM and within voluntary clinical teams.

#### Theoretical framework

**Fig. 1:**
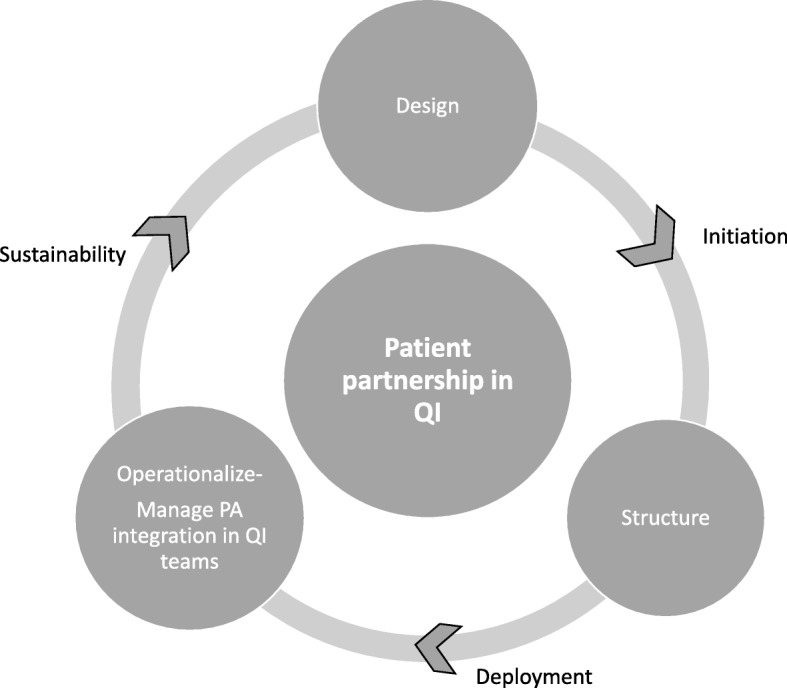
Managerial practices for PP implementation in QI

To organize the collection and the analysis of the data, we built a theoretical framework based on two pieces of literature: managerial work within HCOs and organizational change management (see Fig. 1).

To analyze the process of PP implementation, we relied on studies by Mintzberg and Cloutier and Denis. The work of Cloutier, Denis et al. [29], based on the concept of “institutional entrepreneur”, proposes a typology of managerial work: the work of structuring change, the work of conceptualizing change, the work of operationalizing change, and the relational or sense-making work in the context of change. Mintzberg’s empirical work on managerial roles within organizations [30] highlights ten roles for managers, including relational and information roles with other members of the organizations as well as decision-making roles, such as initiating and designing the change in the organization, and controling the allocation of resources to structure the change. Based on these works, we decided to focus on three sets of managerial practices: 1-*Design practices* refer to managerial efforts to establish new beliefs systems, norms and PP interpretative schemes; 2-*Structuration practices* correspond to managers’ work to formalize the roles of actors involved in PP implementation, as well as to establish the goals, rules, and principles for organizing PP and allocating corresponding resources; 3-*Operational practices* consist of daily managerial actions to manage patient integration within QI teams, including information and relational actions undertaken by managers. Several change phases were studied, inspired by the well-known organizational change management framework from Kotter [31]. Kotter’s framework proposes an eight-step process for leading change in organizations. We decided to group these steps into three main categories. *Initiation* refers to identifying and communicating the need for change, building a guiding coalition, defining a strategic vision of change and enlisting volunteers [31]. *Deployment* corresponds to the implementation of activities to enable change and generate short-term wins [31]. *Sustainability* refers to the ability to institute and sustain change [31]. Our theoretical framework was chosen because it helps identify very succinctly the main types of practices that can be used by managers in the different phases of change management. Due to its simplicity, this framework allows for the emergence of other types of management practices from qualitative data.

### Data collection

Thirty-eight in-person interviews (20 in case 1 and 18 in case 2) were carried out with managers involved in the implementation of PP in QI at three different management levels (see Table 1). We decided to interview managers at different levels to gain a wider perspective of managerial efforts across the entire organization to integrate PP in QI.

**Table 1:** Data collected for each case and data collection period

Type of data	Cases	Period 1 (2015)	Period 2 (2016)	Period 3 (2017)	Total
Interviews with managers	Case 1 (mental health)	Top-level (3) Mid-level (4) Front-line (2)	Mid-level (2) Front-line (1)	Top-level (3) Mid-level (3) Front-line (2)	20
	Case 2 (oncology)	Top-level (3) Mid-level (3) Front-line (2)	Mid-level (2) Front-line (1)	Top-level (3) Mid-level (2) Front-line (2)	18
Total					38
Focus groups with patients and providers	Case 1	Patients (1) Providers (1)		Patients (1) Providers (1)	4
	Case 2	Patients (1) Providers (1)		Patients (1)	3
Total					7

Several managers were interviewed more than once between period 1 and period 3 because they continued to be involved in the implementation of PP initiatives. In total, 14 managers were interviewed in case 1 and 12 in case 2.

Interviews that were conducted lasted between 40 and 60 min and took place during three data collection periods. Top-level management includes the CEO (chief executive officer), the executive management, and the board of directors. Mid-level management includes various positions of senior and middle managers working within an administrative or a clinical department. Finally, front-line management comprises program managers in charge of supervising clinical teams. We also conducted focus groups with patients and providers during two data collection periods (2015, 2017) to gain a wider perspective on the involvement of patients in QI activities and interactions with managers and providers. The focus groups with patients were conducted with different participants in period 3 and involved between 4 and 6 participants. Other focus groups were conducted with providers who were part of the QI teams selected for the case studies (see details of QI teams in Table 2). Semi-directed interview guides were adapted and used for both interviews (see Additional file 1) and focus groups. All interviews were conducted and transcribed in French, then translated into English by a professional translator. To confirm and complete interviews with managers, we collected management documents related to the implementation of PP. Examples of the documents collected are: the strategic plans and the code of ethics of the HCOs, the HCOs’ QI policies, the reference frameworks for PP, the minutes of quality improvement committee meetings.

**Table 2:** Summary profile of the cases

Characteristics of the cases	Case 1	Case 2
Type of HCO	HCO 1 Integrated university health and social services center	HCO 2 Integrated university health and social services center
Location	Rural setting	Urban setting
Initial models of PP	Partners in Care Program	Partners in Care Program
Clinical settings	Mental health	Oncology
Clinical units	Ambulatory hospital services in mental health	Acute services, breast cancer
Composition of QI teams	Program manager, psychiatrist (medical chief), psychologist, occupational therapist, nurse, two PAs, two ICLs	Program manager, radiation oncologist (medical chief), oncologist surgeon, psychologist, two PAs, one ICL
Examples of QI activities with PAs	At clinical team-level: improving patient pathways within ambulatory mental health services; assessing daytime hospital services; adapting physical activities to patients’ needs	At clinical team-level: developing educational activities on life after breast cancer; integrating PAs to facilitate pre-surgery classes for breast cancer, developing strategies to promote educational activities on breast cancer
	At other levels: developing an information platform for wait times; kaizen to review process and tools for recruiting PAs; facilities development projects	At other levels: developing educational activities for patients with cancer, improving the cancer care and services continuum

This study was approved by the UofM’s Health Sciences Research Ethics Committee (certificate #14–127-CERES-D).

### Data analysis

To analyze qualitative data, three successive phases combining a deductive and inductive analysis were conducted [32] using QDA Miner Lite software: 1-data codification and categorization based on an a priori template of codes [33]. In this deductive phase of analysis, a concept-driven coding approach was used since the a priori template of codes was developed from our theoretical framework; 2-identification of new codes and categories following a data-driven analysis and inductive phase of analysis [34]; 3-formulation and validation of our findings with key stakeholders. The first three interviews were coded independently by two reviewers (NC, MPP). Divergent codifications were discussed until both reviewers reached a consensus leading to a coding tree. Management documents were also integrated into the same database and coded using QDA Miner Lite software. Case stories were written to synthesize PP models and the implementation process focusing on managerial practices (NC). For data verification and refinement purposes, we presented our case studies during two knowledge transfer activities (2015, 2016) intended for HCO managers who had participated in the study (NC, MPP). In 2017, we finally summarized and sent our overall results to key managers for each case and incorporated their feedback into our final results (NC).

## Results

Our findings are presented in two sections. For each case, we present a brief synthesis of the implementation of PP initiatives from initiation to sustainability phases. Then, we focus on key managerial practices used to implement PP in QI.

### Implementation of PP initiatives in the two cases

Both cases implemented the Partners in Care Program in 2013 within voluntary clinical teams and benefited from methodological support from UofM during the first year. HCO 1 implemented the program in the ambulatory mental health hospital services unit (case 1), and HCO 2 tested it with its breast cancer unit. Table 2 provides a summary profile of each case and Tables 3 and 4 synthesize the key events that happened between initiation and sustainability of PP in cases 1 and 2.

**Table 3:** Key events between initiation and sustainability of PP

	Case 1 – Mental Health
Initiation 2013–2014	• Initiation of Partners in Care Programs in two clinical teams • Initiation of a large-scale partnership approach in the HCO • Department of research and professional practices responsible for implementing the PP • Recruitment of a PA to help the department structure and implement PP activities
Deployment 2015	• Development of a reference framework for PP and elaboration of a logic model to organize the integration of PAs in QI • Development of a five-step process for involving PAs in QI • Presentations on PP made at different levels of the HCO and explanatory documents of the PP approach • Mental health team completed two QI cycles with two PAs and support from UofM
Sustainability 2015–2017	• HCO merged with eight other HCOs following Quebec healthcare system reform • CEO decided to continue and adapt the PP approach in the new HCO • Quality department responsible for implementing PP • Mental health team completed seven QI cycles with PAs and 200 PAs involved in several QI activities at different levels within the HCO

**Table 4:** Key events between initiation and sustainability of PP

	Case 2-Oncology
Initiation 2011–2013	• Launch of major projects on collaborative practices within the HCO • Strategic committee set up to plan collaborative projects and four clinical teams selected to initiate the Partners in Care Program • Department of multidisciplinary services responsible for implementing PP
Deployment 2014–2015	• Community of practice created to support the initiation of the Partners in Care Programs within clinical teams • Breast cancer team completed one QI cycle with PAs and support from UofM • Involvement of PAs in the co-construction and co-presenting with providers of educational activities for patients
Sustainability 2015–2017	• HCO 2 merged with seven other HCOs • CEO decided to continue the PP approach in the new HCO • Co-existence of two different PP approaches in the merged HCOs • Two successive departments in charge of PP implementation (public health then quality department) • Breast cancer team completed four QI cycles and 10 PAs involved in QI activities within the oncology program

### Managerial practices for the implementation of PP

When implementing PP in QI, managers used four main types of practices: 1-designing the initiative so that PP makes sense to the entire HCO; 2-structuring the initiative to support PP deployment and sustainability; 3-managing PA integration in QI to avoid tokenistic participation of PAs; 4-evaluating PA integration in QI to support the continuous improvement of PP.

#### Designing PP so that it makes sense to the HCO

Designing PP involves making sense of the PP initiative relative to the entire HCO as well as creating a shared vision of PP among managers, providers and PAs. In cases 1 and 2, top (CEO, executive management) and mid-level managers (in the department in charge of PP) integrated the PP approach into the code of ethics, positioning PP as one of the guiding principles of care and services. In both cases, the vision of PP was influenced by external requirements on patient engagement in HCOs, including Canadian accreditation standards and the objectives of the Quebec Ministry of Health and Social Services. Top and mid-level managers contributed to design a PP model that fostered its initiation (cases 1&2) and sustainability in a context of organizational change (case 1). In both cases, top-managers had to maintain an organizational vision of PP and reframe it to suit new organizational structures and responsibilities. In case 2, following the merger of HCOs, the lack of a clear vision from top-level managers, along with insufficient alignment between top-level, mid-level and front-line management regarding PP, compromised the sustainability of the PP model.

*We’re in a strategic blur, a dense tactical fog, and operationally, we all do our own thing with the limited resources we have [ … ] what we lack is a common project, support from upper management* (mid-level manager, case 2)

Case 1 used a range of design practices. First, mid-level managers developed a reference framework for the PP model which clarified the definition of partnership concepts. Furthermore, the mid-level manager in charge of PP, as well as the CEO, in tandem with a PA, gave several presentations about the PP approach to different clinical and management committees. The promotion of the PAs’ role and contribution in QI helped raise awareness of the added value of PAs and encouraged QI teams to involve PAs. In this regard, managers, in co-leadership with PAs, acted as ambassadors and disseminators of the partnership approach.

*We received training on the concept of patient partners with all managers. Patients came to share their stories; we, as care staff, do not always have the patients’ perspective on the services we provide. It raised awareness among all managers. Then, the quality team started including patients, so everyone was on the same page, ready to welcome them* (program manager, case 1)

Additionally, case 1 succeeded in ensuring the transfer of PP experience and knowledge among managers. Continuity among mid-level managers overseeing PP was determinant in the context of a merger to help sustain a vision of the PP model. One of the mid-level managers previously involved in implementing PP in the former HCO was able to share her knowledge and experience about PP with her team.

#### Structuring PP to support its deployment and sustainability

Top and mid-level managers played a key role in structuring the PP initiative to ensure its successful deployment and to facilitate its sustainability over the long term. By structuring PP, managers acted as entrepreneurs in contributing to define the PP model. In both cases, executive management integrated PP into the strategic goals of the HCOs and appointed a department at the mid-level to oversee PP as a core function of the governance structure. In case 1, PP has been defined as contributing to a high quality of care and services.

*That year, we integrated the partnership into the strategic objectives of our new organization. We want to place the whole notion of partnership at the heart of care and service quality* (CEO, case 1)

In cases 1 and 2, different departments (professional practices/multidisciplinary services and quality departments) have been successively responsible for implementing the PP initiative. Structuring PP also required that mid-level and front-line managers organize the coordination of PP activities within the HCOs. In case 1, the coordination of PP was centralized in the quality department where mid-level managers developed a five-step process for PA involvement in QI: 1) PA request from a manager or a provider; 2) verification of the appropriateness of the request; 3) PAs’ identification and recruitment; PAs’ and QI teams’ training; 4) contact of the PA by the manager or provider to explain the QI project and the PA’s role; 5) assessment of the PA’s participation within the QI project. On the other hand, in case 2, the coordination of PP was less formalised and was shared between the breast cancer program manager and the mid-level manager.

In both cases, the mergers destabilized the coordination of PP. In case 2, in the absence of effective governance of PP at top and mid-level management, several mid-level and program managers – previously involved in coordinating the PP activities in their former HCOs – have created a community of practice to foster the harmonization of the PP practices (e.g., PA recruitment, PA satisfaction assessments) in all clinical programs.

*[ … ] as part of our partnership office, to better coordinate our actions, exchange tools and methods developed as much in hospital X as in hospital Y. We try to harmonize, we revised the patient request form and the patient satisfaction form* (program manager, case 2)

In case 1, mid-level managers questioned the future role of the quality department regarding the coordination of all PP activities. As the number of PAs involved in QI has significantly increased in the merged HCO, mid-level managers have experienced challenges in maintaining personalized support for recruited PAs. They suggested that clinical programs could also take part in the coordination of PP, for instance, by creating a list of potential PAs, as well as PA recruitment and preparation.

*Having personalized management for patient banks seems hard to maintain in such a large territory. I’m eager for us to think about this, because if we manage to reach 150 patients, I’m not sure that all patients will receive the same relationship and involvement quality* (mid-level manager, case 1).

Furthermore, structuring PP required middle managers to secure funds to compensate PA participation in QI activities (travel expenses, parking tickets, lunch), to ensure the recognition of PA involvement in QI and to encourage their ongoing participation. In case 2, mid-level managers questioned the sustainability of PA participation in QI activities given the absence of funding to compensate their work.

*For highly involved people, who do more than volunteer, it would be fair to be able to remunerate them, but for now, we lack the structure and funding that enable us to do this. We have a budget from the X Foundation which allowed us to compensate patients who participated in activities, but this budget is running dry* (mid-level manager, case 2)

### Managing PA integration to avoid tokenistic patient involvement

Managing PA integration in QI activities requires managers to select, recruit, prepare and coach PAs, to train providers/managers and to support their collaboration with PAs. These practices represent renewed practices that managers have developed over time to ensure the successful integration and involvement of a new actor (the PA) within QI teams. This range of new practices differs from their usual daily work, including adaptations of typical human resource practices geared towards a rather “unusual” human resource (the PA), especially in terms of selection, recruitment, preparation as well as training and coaching. These practices are carried out by mid-level and front-line managers and are a result of a new type of relationship between managers and patients, who interact on a regular basis for QI purposes.

#### Selecting, recruiting and preparing PAs

Selecting, recruiting and preparing PAs are new practices that managers have developed over time to ensure the successful integration of PAs into QI teams. In case 1, managers systematically verified the appropriateness of involving PAs in a QI team before starting the selection process, to make sure that the QI project reflected patient concerns and that the PAs would add value. In both cases, program managers, in collaboration with providers, identified potential PAs while mid-level managers handled recruitment and preparation of the new PAs. In case 1, the middle-manager benefited from the support of an expert PA in these activities.

For both cases, PA recruitment was done through face to face interviews based on a set of core skills expected from PAs as set out by UofM. These skills are: having experienced services related to the QI committee; a stable health condition; effective communication; availability to participate in several meetings. In both cases, PAs were trained on PP principles and objectives, as well as PA roles and responsibilities on a QI team.

*It was actually helpful to learn more about what a partnership is, what we can bring to the table as a patient, what our role and responsibilities are when we are involved in a team* (PA, focus group with patients, case 2)

In case 2, providers reported the need to clarify PA roles and responsibilities, for instance, regarding access to and handling of confidential information.

*Preparation is provided to new patients; it’s a must. X and team did that. Because patients arrive in good faith, yes, but sometimes there are things that are important for them to know. Like, the extent of their role. At least the notion of confidential information* (program manager, case 2)

#### Team training

For both cases, during trials of Partners in Care Programs, QI teams were first trained on partnership concepts and methods by UofM. A specific effort was made to explain the roles and responsibilities of new team members, including PAs and ICLs. For both managers and providers, it was necessary that QI teams be trained and prepared to work in co-construction with patients before integrating PAs.

*A major success factor is the thorough work that goes into preparing providers and patients before getting started, with help from UofM* (top-manager, case 1)

*The whole training that we had with UofM, I think, was very helpful, because we didn’t know much about partnership, what the role of PAs would be within our team* (physician, focus-group with providers, case 2)

At the end of Partners in Care Program trials, each case adopted different practices to ensure QI team training. In case 1, the middle-manager, in partnership with an expert PA, systematically provided individual training for QI teams interested in partnering with PAs as well as an explanatory document containing information on PP principles, benefits and processes to be followed for PA integration. Involving a PA as a co-trainer helped the QI teams recognize the added value of partnering with patients in the QI process.

*We always provide a training tandem: a Quality Advisor and a PA for new PAs and teams that want to integrate PAs. This tandem is a must!* (mid-level manager, case 1)

*In this presentation, I remember that the patient shared her story, her experience as a patient. We realized that patients have valuable things to share with us to improve the services that we offer them* (psychiatrist, focus-group with providers, case 1)

In case 2, the mid-level manager created a community of practice, bringing together all QI teams that partnered with PAs. This community helped share experiences, practices, methods and issues related to PA integration, as well as develop a charter on good PP practices and methods.

#### Supporting collaboration between PAs and QI teams

Integrating PAs into QI teams required daily efforts from mid-level and front-line managers to support and stimulate collaboration among PAs, providers and/or managers. During the trials of Partners in Care Programs, ICLs helped program managers act as PP facilitators and helped ensure that PAs and QI teams mutually understood their roles and responsibilities. Meanwhile, the program manager also had to facilitate compromise when setting QI objectives so as to satisfy the concerns and expectations of PAs, providers and managers.

*There is certainly a gap between my perceptions and concerns as a manager and those of professionals and patients. Our challenge is to find an objective that will connect everyone’s interests, particularly those of the patients if we want them to be involved* (program manager, case 2)

In both cases, mid-level managers set rules to facilitate PA participation and their integration into teams: involvement of at least two PAs on QI teams; and assignment of a patient coach, who has PA experience, for newly recruited PAs. PAs appreciated the ongoing support of a patient coach. This coach encouraged PAs to express themselves and to share their expectations which helped their meaningful involvement on QI teams.

*He gave me good advice to make me feel more comfortable expressing my opinions and expectations with the team, which I appreciated* (PA, focus-group with patients, case 1)

Program managers faced issues related to continuous PA involvement on QI committees. In case 1, the program manager struggled with high PA turnover on the committee, for several reasons (medical condition, work). Finally, one particular issue was raised by the mental health QI team in relation to PA support once their involvement ends. For patients with mental health issues, participating in QI teams as a PA also represents a step towards recovery. Therefore, ending their involvement could be badly experienced if their exit is poorly prepared and if the PA transition is not supported by the team.

*For patients, project or team involvement means a lot for their recovery as it becomes a benchmark for therapeutic success or failure, even though it is a collaborative relationship. I believe that PAs should be supported at the end of their involvement or shepherded in terms of what the end of their involvement means* (psychologist, focus group with providers, case 1)

### Evaluating PA integration in QI to support continuous improvement

In case 1, mid-level managers from the quality department collected data to report on PP integration in QI activities. Collected data included: number of PA requests in QI; types and number of departments, programs or clinical teams involving PAs in QI; and different PA involvement purposes. Collecting these data helped case 1 to continuously monitor PP activities across the whole HCO. In case 2, a systematic collection of these types of data was not implemented in the quality department.

On the other hand, in order to ensure continuous improvement of PP, evaluating PP in QI mainly involved assessing the PA integration process by understanding how PAs and QI teams experienced their partnership. In both cases, this informal practice was carried out by program managers who inquired about PA satisfaction in terms of team integration and participation, and of potential areas of improvement. They regularly shared PA feedback during team meetings and, in turn, providers shared their own partnership experiences with PAs.

*We have meetings with patients and professionals to assess participation – what went well and what went less well – so that everyone provides their opinion* (program manager, case 2).

In case 1, since the coordination of PP was centralized in the quality department, the role of mid-level managers was to evaluate PP. The formal PA involvement assessment process relied on three main types of data: PA satisfaction with regard to their participation; PA benefits gained from their partnership experience; QI team members’ perceptions regarding PA contributions and PA partnership challenges. This formal assessment process helped the quality department to sustain continuous improvement of the PAs’ integration and to support QI teams that face specific challenges with PAs.

**Fig. 2:**
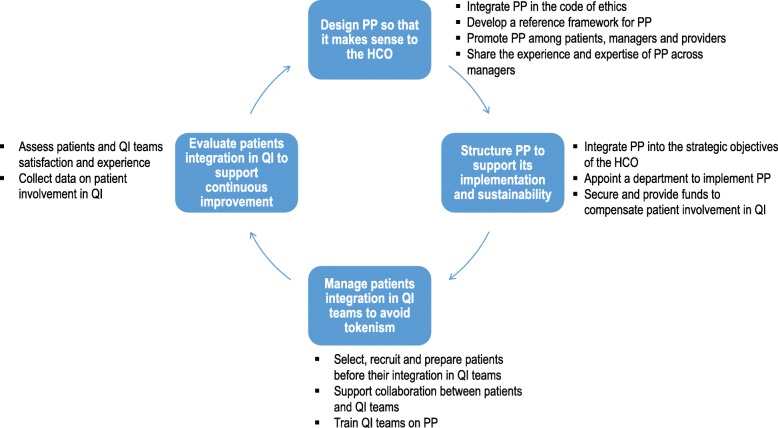
Synthetic presentation of the main findings

A summary of the findings is presented in Additional file 2 and a graphic presentation of the main conclusions is provided in Fig. 2.

## Discussion

To our knowledge, this is the first study to focus on key practices of HCO managers when implementing PP in QI. Thanks to our framework, we have shown that managers are engaged in four main types of practices: 1-designing PP so that it makes sense to the entire HCO; 2-structuring PP to support its deployment and sustainability; 3-managing PA integration into QI to avoid tokenistic involvement; 4-Evaluating PA integration into QI to support continuous improvement. The two first types of practices are based on typical practices, used by managers to implement change initiatives in HCOs, whereas managing and evaluating PA integration require new daily practices that managers did not necessarily rely on before. Our results have also revealed that managers, at all management levels, are concerned with implementing PP in QI, from top to front-line managers.

### Designing and structuring PP: how managers adapt typical change management practices?

Our research indicates that top and mid-level managers have contributed to defining the ways in which PP was designed and structured within HCOs. These managerial practices helped initiate PP and sustain them over time. In fact, most of the practices used by managers to implement PP are usually adapted from those used to implement change initiatives within HCOs, such as the introduction of QI initiatives.

The literature on change management and QI implementation highlights key elements that ensure successful implementation of new initiatives within HCOs, including: the adoption of frameworks regarding the change to be introduced [35–37]; the promotion of change initiatives [38–40]; and the capacity to transfer knowledge on change initiatives among managers [41, 42]. Creating a shared vision of QI is one of the main spheres of activities specific to quality management in HCOs [35, 43]. Our study shows that managers play an important role in designing PP so as to create a shared vision of the model. To do so, they integrated the PP into their codes of ethics (cases 1&2), developed a reference framework for the PP model (case 1), promoted PA contributions in QI (case 1), and ensured that PP implementation knowledge and experience were shared among managers (case 1). HCO managers contributed to a cultural change toward PP in QI. Cases 1&2 illustrate how commitment from top-level managers and aligning the vision across management levels can influence an initiative’s sustainability [37, 44], especially within the context of a merger [45, 46]. They also acted as PP ambassadors and disseminators in order to encourage QI teams to integrate PAs.

QI implementation studies also recognize the key role of HCO managers in setting QI goals [47], centralizing QI goals and tasks as a core function of the governance structure [43], and supporting QI initiatives via adequate resource allocation [48–51]. These key practices were used by top and mid-level managers to support PP implementation and its sustainability over time. Structuring PP included a broad range of practices, namely: integrating PP initiatives into the HCOs’ strategic goals (cases 1&2), appointing a department in charge of PP (cases 1&2), developing a logic model, specifying goals, targets, strategies to implement PP (case 1), providing sufficient funds to compensate PAs (cases 1&2). In that regard, top and mid-level managers act as entrepreneurs setting the course of change and, as resource allocators, supporting change and making it achievable [30, 52].

While managers adapted typical change management practices to support the implementation and sustainability of the PP initiatives, they also faced new challenges that would need to be addressed in the future and have not been studied so far. With PA involvement growing within HCOs, managers have called for a deeper analysis of how to coordinate the PP activities, the future role of clinical departments, as well as the allocation of dedicated resources to ensure ongoing PA involvement in QI activities [53].

### Managing and evaluating PP in QI: towards renewed practices for healthcare managers

Our study shows that mid-level and front-line managers were engaged daily in implementing PP in QI, which involved managing and evaluating PA integration into QI teams. Those practices included: selecting, recruiting and preparing PAs; supporting collaboration among PAs, providers and managers; team training and PA integration assessments. This range of practices results from a new type of relationships between managers and patients, who interact on a regular basis for QI purposes. Managers had to develop renewed practices to ensure successful integration of a new actor (the PA) into QI teams, avoid tokenistic PA involvement and support continuous improvement of their engagement. This range of new practices differed from the managers’ daily work, and some practices were viewed as adaptations of typical human resources practices for a rather “unusual” human resource (the PA), especially in terms of selection, recruitment, preparation, training and coaching.

Moreover, several studies on factors enhancing patient engagement in QI support some of these findings, including the need for structured methods to select and recruit PAs, to train both PAs and teams [12, 18, 49] in order to avoid tokenistic patient engagement [20, 54]. Program managers are engaged daily in facilitating PA integration into QI teams and supporting collaboration between PAs and other QI team members. Their role as PP facilitation agents is important. Identifying clear roles and responsibilities for PAs [5, 20], choosing the right PAs relative to the QI mandate, and assigning a patient coach to PAs [12, 53] can foster effective PA involvement in QI teams. In mental health, specifically, program managers must continuously support PAs since their involvement in QI teams may also represent a step towards clinical and personal recovery.

Lastly, managers are engaged in evaluating PA integration into QI. In case 1, mid-level managers formally assessed PP activities by collecting process (satisfaction and experience) and structural indicators. In both cases, program managers evaluated PA and team satisfaction and experience through regular feedback. This informal evaluation with all team members is essential to reflect and suggest ways to continuously improve PP [12]. Managers must also measure the impact of PP on quality, which represents a challenge for any patient engagement initiative in QI [5, 55, 56].

### Managers’ contribution to expanding PA involvement in QI within HCOs

Our findings shed light on how HCO managers contribute to shaping and expanding PA involvement in QI over time within HCOs. Both study cases initially tested two similar programs at the clinical level. Thanks to managerial efforts, PA involvement expanded beyond clinical QI teams, especially in case 1, in which top-level managers cooperated with mid-level managers to successfully develop a shared vision of a large-scale PP model in QI. This resulted in PA integration within various HCO organizational committees, projects, programs and QI areas [53]. Mid-level managers were able to share their PP vision with front-line managers and QI teams while helping them achieve PA integration into QI activities thanks to structured processes. In case 2, mid-level managers, in collaboration with front-line managers, also integrated PAs into QI activities within the oncology program, namely in the development of educational activities for all patients treated for cancer. As suggested by the literature related to quality improvement, cooperation among managers – across different management levels – is a critical element for implementing, expanding and sustaining new initiatives within HCOs [43, 57].

### Limits of the study

One of our study’s limitations was the impact of HCO mergers following the provincial health system reform, which hindered research on the sustainability of PP initiatives in a stable organizational context. However, it turned out to be an opportunity to understand how managers, at different levels within HCOs, contribute to maintaining and adapting or not a PP model in a context of organizational change. Although our study is based on a longitudinal design, since we collected data during three periods, it should be noted that the data collection started few years after the beginning of PP initiatives in the two cases. However, the two last periods of data collection took place during the implementation of PP in both cases (2015 to 2017). Finally, our research focused on PP implementation in QI, which is a specific model of patient engagement in QI and was also limited to two different clinical teams and HCOs. Future research should be undertaken on HCO management practices for the implementation of a broader range of patient engagement initiatives and within various clinical and organizational settings.

## Conclusion and implications for HCO managers

This research adds to the evidence of the daily role plays by HCO managers when implementing PP in QI and it contributes to guiding HCO managers through the integration of patient engagement initiatives. Implementing PP requires renewed practices for HCO managers, which can be challenging. While external requirements encourage HCOs to partner with patients in QI, managers need to be supported through specific training on best practices for managing PP and various forms of patient engagement in QI. The Faculty of Medicine at UofM has recently developed an online course on PP, which is offered to students at different medical sciences and health administration programs to help them work with PAs [58]. While this initiative is interesting, exhaustive training should also be offered in academic institutions, such as in schools of public health, nursing schools, and schools of management.

## Supplementary information

**Additional file 1:** Selection of interview questions with managers.

**Additional file 2:** Managerial practices used at different management levels to implement patient partnership in quality improvement.

## Data Availability

The data (quotes) generated during the current study are available from the corresponding author on reasonable request. Some data (selection of quotes) are shared in the results section.

## Abbreviations

CEO: Chief executive officer
HCO: Healthcare organization
ICL: Institutional collaboration leader
PA: Patient advisor
PP: Patient partnership
QI: Quality improvement
UofM: University of Montreal
