# Supplementary material for: Partnering with patients in quality improvement: towards renewed practices for healthcare organization managers?
Source: BMC Health Serv Res. 2019 Nov 8;19:815. doi: 10.1186/s12913-019-4618-8 (PMC6839263; doi:10.1186/s12913-019-4618-8)
Supplement: Supplementary file 1 — Additional file 1: Selection of interview questions with managers. [file 12913_2019_4618_MOESM1_ESM.pdf]

Additional file 1: a selection of interview questions with managers

1- What is your function in the organization?

2- Can you describe the patient partnership approach in quality improvement that has been implemented in your organization?

3- In which quality improvement activities or teams are patient advisors involved?

4- What is your role in implementing the patient partnership approach, in integrating patient advisors in quality improvement activities or teams?

5- What are your tasks, daily activities related to this approach?

6- With whom (department, program, type of managers) do you collaborate to ensure these activities?

7- What challenges do you face as a manager to implement this approach and ensure the management and the evaluation of these activities?
