# Supplementary material for: Partnering with patients in quality improvement: towards renewed practices for healthcare organization managers?
Source: BMC Health Serv Res. 2019 Nov 8;19:815. doi: 10.1186/s12913-019-4618-8 (PMC6839263; doi:10.1186/s12913-019-4618-8)
Supplement: Supplementary file 2 — Additional file 2: Managerial practices used at different management levels to implement patient partnership in quality improvement. [file 12913_2019_4618_MOESM2_ESM.pdf]

Additional file 2: Managerial practices used at different management levels to implement patient partnership in quality improvement

Type of managerial practices	Key practices of managers at different levels
**Designing PP**	**Top-level managers:**<br>Adopt and maintain an organizational vision of PP (case 1)<br>Integrate PP in the code of ethics (case 1)<br><br>**Top-level and mid-level managers:**<br>Develop a reference framework of the PP model (principles and associated values) (case 1)<br>Include PP in the code of ethics (cases 1&2)<br>Promote PP among patients, providers and managers and raise awareness about the contribution of PA in QI (case 1)<br>Ensure alignment between top-level, mid-level and front-line managers regarding the PP model (case 1)<br><br>**Mid-level managers:**<br>Transfer PP experience and knowledge among managers (case 1)
**Structuring PP**	**Top-level managers:**<br>Include PP as part of the HCO's strategic goals (cases 1 & 2)<br>Appoint a department and managers in charge of PP (cases 1 & 2) and ensure continuity among the mid-level managers in charge of PP (case 1)<br><br>**Mid-level managers:**<br>Provide sufficient funds to compensate PA involved in QI activities (case 1 & 2)<br>Involve a PA to collaboratively structure the PP model (case 1)

	Develop a logic model of PP (goals, targets and strategies to deploy PP) and review it over time (case 1)<br>Coordinate a structured PA integration process in QI (case 1)<br>Create and manage a list of potential PAs (cases 1&2)
**Operationalizing PP<br>-<br>Managing PA integration in QI teams**	**Front-line managers:**<br>Identify potential PAs with support from providers (cases 1&2)<br>Stimulate and support the partnership among PAs, providers and managers (cases 1&2)<br>Facilitate compromise when setting QI objectives (cases 1&2)<br><br>**Mid-level managers:**<br>Recruit and prepare PA before their integration in QI activities (cases 1&2)<br>Provide individual training to QI teams and explanatory documents on PP methods (case 1)<br>Create a community of practice and a chart for good PP practices (case 2)<br>Set rules to facilitate PAs participation and integration in QI teams (cases 1&2)<br><br>**Mid-level managers and front-line managers:**<br>Create an informal committee to foster the harmonization of PP practices in the context of a merger (case 2)
**Evaluating PA integration in QI teams**	**Front-line managers:**<br>Ensure feedback from PAs and QI teams regarding their partnership experience (cases 1&2)<br><br>**Mid-level managers:**<br>Formally evaluate the PA integration process (case 1)<br>Collect structural indicators on PP activities (case 1)
